# Haemorrhage control of the pre-hospital trauma patient

**DOI:** 10.1186/1757-7241-22-S1-A4

**Published:** 2014-07-07

**Authors:** Ross Davenport

**Affiliations:** 1 Centre for Trauma Sciences, Blizard Institute, Queen Mary University of London, UK

## 

Haemorrhage following major injury remains the most common potentially preventable cause of traumatic death with exsanguination typically occurring within three hours of injury. Key to improving outcomes is a “care bundle” of measures to facilitate early diagnosis, rapid haemorrhage control, systemic and topical haemostatic support and short scene times [1]. The last decade has seen a paradigm shift in treatment strategies and transfusion algorithms [2] with an emphasis on haemostatic or damage control resuscitation (DCR). A key objective of this proactive and empiric approach to transfusion is to directly target Acute Traumatic Coagulopathy (ATC) which itself is associated with a four-fold increase in mortality and poor patient outcomes. This article will focus only on advanced interventions without further discussion of the fundamentals of pre-hospital haemorrhage control i.e. oxygenation, intravenous or intra-osseous assess, pelvic and limb splints and splinting of the facial skeleton.

Identification of major haemorrhage must be performed as part of the initial on-scene assessment. Good clinical acumen and a high index of suspicion remain important diagnostic tools. Point of care measures of coagulation are of limited value in patients with major haemorrhage (low haematocrit). The utility of these handheld devices, in their current design, is confined to patients taking warfarin who have suspected traumatic brain injury and where reversal with prothrombin complex concentrate is indicated. Pre-hospital use of ultrasound, solely for diagnostic purposes, remains an area of controversy. Critiques argue it adds little additional information and may delay transfer to hospital for definitive haemorrhage control.

An immediate priority is to localise any sites of external haemorrhage and provide direct control where possible with compression. Foley catheters inserted into a wound track, inflated and then retracted to the skin edge are good adjuncts for junctional injuries e.g. neck stab wound. Topical haemostatic dressings e.g. Celox impregnated gauze or granules are able to augment clot formation and can be applied into cavities where haemorrhage may be difficult to control directly. The use of limb tourniquets is now well established in combat medicine and has been shown to save lives particularly when applied before the onset of shock. Complications i.e. nerve damage are rare when tourniquets are applied correctly. The Eastern Association for the Surgery of Trauma (EAST) has recently published guidelines advocating their use in civilian patients for the management of penetrating lower extremity arterial trauma [3].

Radical interventions for control of life-threating haemorrhage include prehospital thoracotomy (PHT) for cardiac tamponade and (theoretically) resuscitative endovascular balloon occlusion of the aorta (REBOA) for major torso bleeding. PHT for penetrating trauma is an established intervention in a physician-led EMS system and is associated with survival rates of 18% in selected patient groups. Relief of tamponade with control of cardiac injury is the primary objective of PHT and can be achieved with minimal equipment – scalpel, Spencer Wells forceps, heavy scissors or Gigli saw, rib retractor and sutures or skin stapler. During PHT other manoeuvres such as manual compression (or clamping) of the descending aorta to arrest abdominopelvic haemorrhage and control of lung hila to treat great vessel injuries may be of benefit but all efforts should be focused on rapid transit to hospital for definitive surgical management. REBOA describes the insertion of an endovascular balloon into the femoral artery (under ultrasonic guidance) which is advanced into the aorta and then inflated to control distal haemorrhage. It has been successfully used in the Emergency Department (ED) with and without image intensifiers depending on the aortic zone of interest [4]. Although feasible in pre-hospital care it remains subject to proof of concept evaluation although has a potentially important role in the pre-hospital management of major pelvic injuries. Balloon occlusion at the level of the aortic bifurcation can be achieved without the use of fluroscopy and could theoretically control catastrophic pelvic bleeding.

Direct control of haemorrhage must be accompanied with DCR - permissive hypotension, resuscitation and haemostatic support with pro-coagulant blood products. The efficacy of high dose blood component therapy and optimal plasma:red blood cell (RBC) ratio is currently unknown. However, the PROPPR trial (http://cetir-tmc.org/research/proppr) is the first randomised controlled trial (RCT) to investigate blood product ratios in trauma and is due to report in early 2014. Combat pre-hospital systems utilise both RBC and thawed fresh frozen plasma (FFP) but due to the logistical challenges of carrying these blood products the vast majority of civilian EMS systems only utilise RBCs. Blood resuscitation of the exsanguinating patient may be the preferable therapeutic option compared to crystalloids but its pre-hospital use must be supported with rapid availability of plasma, platelets and fibrinogen on arrival in ED to avoid the dilutional coagulopathy associated with large volume RBC transfusion. Work is currently underway to evaluate freeze dried plasma which has the advantage of potential reconstitution at scene however the true efficacy of any plasma formulation to correct ATC remains to be seen.

Haemostasis is critically dependent on fibrinogen as a substrate for clot formation and levels are known to decrease both rapidly and significantly during massive haemorrhage. Low fibrinogen levels are associated with increased mortality and early fibrinogen supplementation (cryoprecipitate or concentrate) may improve patient survival [5]. Early and high dose fibrinogen replacement is the subject of two pilot RCTs in the UK (CRYOSTAT - ED) and Austria (FiTIC – pre-hospital) due to report later this year. Fibrinogen concentrate is well-suited for use in austere environments and has the potential to be the primary pro-coagulant agent for pre-hospital DCR. All severely injured patients with bleeding have profound activation of the fibrinolytic system. The CRASH-2 trial has shown clear mortality benefits with the early use of tranexamic acid (TXA) in trauma haemorrhage, which should be considered first line in all patients with major bleeding.

Effective treatment of haemorrhage in the pre-hospital phase of care is dependent on rapid identification of severe bleeding, direct anatomical control of vascular injury, haemostatic resuscitation and expedient transfer to hospital. Tourniquets and topical haemostats are important adjuncts for the control of junctional or extremity injuries and augmentation of *in vivo* clotting processes. PHT is a life-saving and established intervention for cardiac tamponade and pre-hospital use of REBOA may provide additional treatment options to control major torso haemorrhage. Replacement of oxygen-carrying capacity with RBC, haemostatic resuscitation with fibrinogen supplementation and treatment of fibrinolysis with TXA are likely to prove important therapeutic agents in the prehospital treatment of haemorrhage.
